# Analysis of Head Impact Biomechanics in Youth Female Soccer Players Following the Get aHEAD Safely in Soccer™ Heading Intervention

**DOI:** 10.3390/s21113859

**Published:** 2021-06-03

**Authors:** Victoria E. Wahlquist, Thomas W. Kaminski

**Affiliations:** Athletic Training Research Laboratory, University of Delaware, Newark, DE 19716, USA; vwahlq@udel.edu

**Keywords:** repetitive head impacts, football, concussion, wearable sensors

## Abstract

The effects of repetitive head impacts associated with soccer heading, especially in the youth population, are unknown. The purpose of this study was to examine balance, neurocognitive function, and head impact biomechanics after an acute bout of heading before and after the Get aHEAD Safely in Soccer™ program intervention. Twelve youth female soccer players wore a Triax SIM-G head impact sensor during two bouts of heading, using a lightweight soccer ball, one before and one after completion of the Get aHEAD Safely in Soccer™ program intervention. Participants completed balance (BESS and SWAY) and neurocognitive function (ImPACT) tests at baseline and after each bout of heading. There were no significant changes in head impact biomechanics, BESS, or ImPACT scores pre- to post-season. Deficits in three of the five SWAY positions were observed from baseline to post-season. Although we expected to see beneficial changes in head impact biomechanics following the intervention, the coaches and researchers observed an improvement in heading technique/form. Lightweight soccer balls would be a beneficial addition to header drills during training as they are safe and help build confidence in youth soccer players.

## 1. Introduction

In recent years, the American media has focused its attention on concussions in youth sports and what the potential long-term effects are. Much of this concern stems from the reports of chronic traumatic encephalopathy (CTE) being found in American football players and contact-sport athletes, including some soccer players [1,2,3,4,5]. CTE is clinically presented as behavior or mood changes such as explosivity or depression and/or cognitive impairment such as memory loss or executive dysfunction [1,2,3]. Currently, CTE can only be confirmed through an autopsy with ongoing research working to find biomarkers of CTE [1,2]. CTE was first reported in boxing and then later in American football, ice hockey, rugby, and soccer [2,4]. It is suspected that the head impacts that occur in these sports play a role in the development of CTE. However, it is still unknown exactly how these head impacts are related to CTE.

Soccer, the most popular sport in the world with around 265 million players [6], is unique in that players purposefully use their heads to advance the ball in play. In the United States, soccer is one of the most popular youth sports with 3 million participants [7]. In 2015, the United States Soccer Federation (US Soccer) put forth guidelines on purposeful heading in the youth population as a part of the US Soccer Concussion Initiative. Children 10 years old and under are banned from heading the ball and those 11–13 years old are limited in practice and unrestricted in games. Once players are 14 years old, there are no restrictions on heading [8]. The 11–13-year-old age group is of interest since they are allowed to start practicing purposeful heading and experiencing repetitive head impacts (RHI). RHI are head impacts that do not result in a known concussion and include impacts such as tackles in American football, checking in ice hockey, and purposeful heading in soccer [9].

While the US Soccer Concussion Initiative is laudable, it was introduced with little guidance to member organizations to implement and monitor. Furthermore, coaching strategies to develop instructional activities for safely teaching important purposeful heading skills in youth soccer players were desperately lacking. In fact, results from the recent survey on purposeful heading in US youth soccer players and adherence to the 2015 US Soccer heading guidelines suggest many gaps in the delivery by youth coaches of basic soccer heading skills [10]. Thus, there appears to be a void in the coaching playbook for inserting and implementing early developmental heading skills and techniques for youth soccer players. The Get aHEAD Safely in Soccer™ program [11] was designed to educate youth soccer coaches on how to properly teach the correct techniques for purposeful soccer heading in the 11–13-year-old age group. Furthermore, the program focuses on five principles: (1) teach proper heading technique, (2) develop strong neck and core musculature, (3) avoid dangerous play situations, (4) educate referees who enforce the rules and guidelines, and (5) use lightweight soccer balls to decrease the mass imparted on the head [11]. The program equips coaches with an array of progressing header drills. Coaches are also equipped with strengthening exercises for the neck and core muscles. Research has shown that head accelerations are related to neck strength, where greater neck strength is correlated to lower head acceleration [12,13,14].

An acute bout of heading is utilized by many researchers in order to control the number and rate of headers, as well as the speed of the ball [15,16,17,18]. Self-reported symptoms, postural control, and neurocognitive functioning are commonly measured following an acute bout of heading; they are also widely used in concussion baseline testing [15,16,17,18]. These clinical measures are used to help determine if there are any detrimental effects due to RHI. The high school and collegiate populations have been widely studied as compared to the youth population [15,16,18,19,20]. Furthermore, the prevalence of concussion among female soccer players is higher than their male counterparts, thus there is a need to further elucidate the gaps that exist between the two sexes. The purpose of this study was to examine the effects of an acute bout of heading on balance, neurocognitive functioning, and head impact biomechanics both before and after the Get aHEAD Safely in Soccer™ program intervention. We hypothesize that all measures would improve following the Get aHEAD Safely in Soccer™ intervention.

## 2. Materials and Methods

Female youth soccer players in the Under 12 division (U12) were recruited from a local youth soccer club during one fall season of play (July–November 2018). This study was approved by the university institutional review board (UDIRB 1254306-3). Participants signed informed assent forms and parents/guardians signed informed parental permission forms. A general health questionnaire, which included demographic data, injury history (in the previous six months), concussion history, player position, and number of years played, was completed by all participants. Participants were excluded from the study if they had a history of neurologic disorder, cervical spine injury, or head injury (i.e., concussion) in the 6 months before data collection.

**Figure 1:**
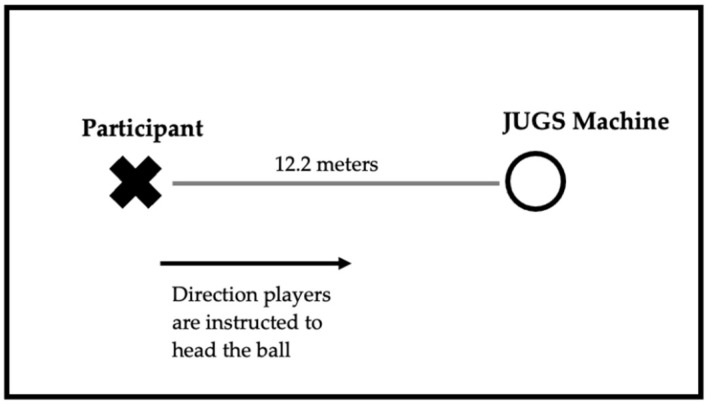
SWAY Balance stances from left to right: Feet together, tandem right, tandem left, single-leg right, and single-leg left.

Participants completed an acute heading protocol, pre- and post-season, at an indoor turf facility. A JUGS soccer machine (JUGS, Tualatin, OR, USA) projected lightweight, size 5 “Header Trainer” soccer balls (The Training Triangle, LLC, State College, PA, USA; roughly 225 g) at 11.2 m/s (25 mph) at a 45-degree angle to the participants who stood approximately 12.2 m (40 feet) away (Figure 1). Participants completed 12 headers in 12 min (one header per minute) and were told to head the soccer ball back towards the JUGS machine. The first two headers were considered a warm-up so the participants could become familiar with the acute heading protocol. The participants were instructed to head the ball given whatever prior experience they had with the skill. We did not offer any technical advice, and if they failed to make contact with the ball, we simply projected another ball their way. During the acute heading protocol, participants wore a Triax SIM-G (Triax Technologies, Norwalk, CT, USA) head impact sensor. Each head impact sensor has a triaxial accelerometer and triaxial gyroscope that measures peak linear acceleration (PLA) and peak rotational velocity (PRV) respectively. Peak rotational acceleration (PRA) is calculated from the rotational velocity. The sensor was inserted into a headband which secured the sensor to the back of the participant’s head. Each participant was fitted with the correct headband size for an optimal, snug fit. PLA, PRV, and PRA were measured during both the pre- and post-season acute heading sessions.

**Figure 2:**
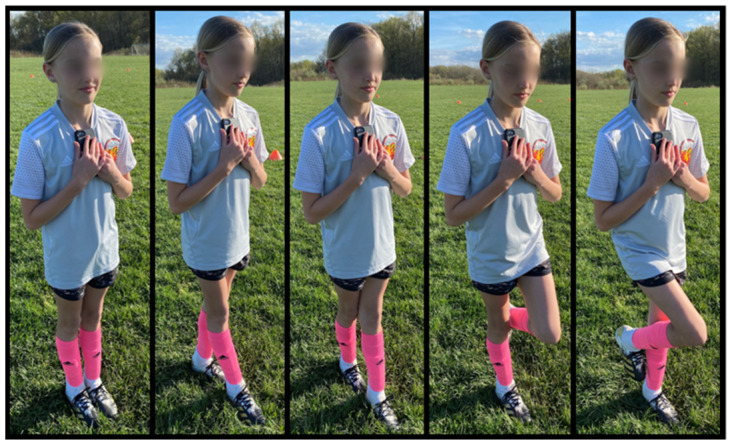
SWAY Balance stances from left to right: Feet together, tandem right, tandem left, single-leg right, and single-leg left.

Participants completed balance and neurocognitive function assessment measures at baseline and after acute heading both pre- and post-season. Baseline measures were taken directly before the pre-season acute heading session. Baseline and pre-season measurements were performed on the same day in July (before and after the acute bout of heading) and post-season measurements occurred 4 months later in November. Balance was evaluated using the Tekscan MobileMat (Tekscan, Boston, MA, USA), Balance Error Scoring System (BESS), and the SWAY Balance application (SWAY Medical, Tulsa, OK, USA). The BESS test includes 3 different stances (double-leg, single-leg (non-dominant), and tandem (dominant foot in front)) completed on a firm and foam surface for a total of 6 tests. Each test was 20 s with the participants’ eyes closed and hands on hips. Participants completed each test barefoot or in socks. The total number of errors (maximum 60) was recorded with a maximum of 10 errors per stance. SWAY is a mobile application that includes 5 different stances for balance (Figure 2) and a reaction time test. Each balance test was 10 s with the participant barefoot or in socks, closing their eyes, and placing the mobile device firmly against their chest. The reaction time test requires the participants to quickly shake the mobile device as soon as the screen changes from a white color to an orange color. SWAY records balance as a percentage of 100 (100% being perfect) and reaction time in ms. The neurocognitive function test utilized was the Immediate Post-Concussion Assessment and Cognitive Testing (ImPACT) computerized test. ImPACT consists of 3 parts: (1) sports and health history, (2) current symptoms and conditions, and (3) neurocognitive testing. The neurocognitive testing involves eight modules that evaluate memory, processing speed, motor functioning, executive functioning, and attention. The outputs of ImPACT are verbal memory, visual memory, visual motor speed, reaction time, and symptom score. Participants completed the ImPACT testing in a quiet room, free from distractions.

During the soccer season, participants took part in the Get aHEAD Safely in Soccer™ program [11]. Before each practice (two times per week), players participated in neck and core strengthening exercises as outlined in the program (Table 1). One time per week during practice, players took part in appropriate purposeful heading drills which included 15–20 purposeful headers. The lightweight, size 5 “Header Trainer” soccer balls were utilized during these purposeful heading drills.

Balance and neurocognitive measures were compared at baseline, pre-season, and post-season using a repeated measures ANOVA. Post hoc dependent samples *t*-tests were used to compare two timepoints when appropriate. Head impacts kinematics were compared using dependent samples *t*-tests.

## 3. Results

Twelve female youth soccer players on a U12 division travel team participated in the study (Table 2). Significant differences in three of the five SWAY stances were observed: feet together, tandem left, and single-leg right. The feet together stance had a statistically significant decrease from baseline to post-season (*p* = 0.009; 98.9 ± 1.9% vs. 96.8 ± 3.0%) and pre-season to post-season (*p* = 0.008; 99.0 ± 1.2% vs. 96.8 ± 3.0%). A significant difference between baseline and post-season for the tandem left stance was observed (*p* = 0.019; 94.3 ± 5.6% vs. 63.9 ± 31.9%), with the score decreasing by 30%. The single-leg right stance also had a significant decrease from baseline to post-season (*p* = 0.020; 72.1 ± 21.8% vs. 41.4 ± 36.1%) with a change of 30%. No differences were observed for self-reported symptoms, BESS, or ImPACT scores (Table 3).

Head impact kinematics were recorded for pre- and post-season acute heading sessions for eight of the twelve players. The remaining four players had no head impact kinematics recorded either pre- or post-season suggesting that the impacts were under the head impact sensor threshold of 10*g*. There were no changes in PLA, PRA, or PRV from pre- to post-season acute heading sessions. Results are presented in Table 3.

## 4. Discussion

There is growing concern in the soccer world around RHI, especially in youth participants. The biggest concern is what the long-term effects of RHI are and what role RHI plays in the development of CTE. This study, using a pre- versus post-test design, set out to examine changes in typical concussion symptomology, neurocognitive function, and balance in a group of skilled, female youth soccer players. Furthermore, we introduced a novel acute heading protocol based on previous models involving launched soccer balls, but in our situation, we employed the use of lightweight regulation-size soccer balls. Our main findings involved subtle changes in three of the SWAY stances at the end of the playing season. There were no significant differences with regards to computerized neurocognitive testing, balance as measured by the BESS, and self-reported symptoms, along with head impact biomechanics.

This study was unique in that we employed a novel soccer heading instructional intervention that included a focus on neck and core strengthening exercises. Particular attention in the program utilized a variety of simple, easy-to-use partner exercises that our youth soccer players quickly accepted and adapted to. Furthermore, feedback from the players was positive, and the coaches appreciated that it was focused and not very time-consuming as to detract from their primary practice schedule. Moving forward, we would suggest continued modification with the sets and repetitions that are suggested in Table 1 to further validate the objective of the neck and core strengthening exercises and teaching effective soccer heading in youth players.

Mixed results on balance and postural control outcomes following soccer heading have been previously reported [15,16,19,20,21]. Following an acute bout of heading, no changes in balance were observed in male and female collegiate soccer players (aged 18–21 years) [15,19]. Both of these studies involved an increased number of headers and both linear and rotational headers as compared to the current study which only involved linear heading. Kaminski et al. [15] utilized the BESS test and Broglio et al. [19] utilized a force plate with a visual conflict dome to measure balance. Our study utilized both the BESS test and the SWAY Balance application. Haran et al. [20] reported changes in postural control in collegiate soccer players (aged 19–26 years) after an acute bout of heading. Their heading protocol setup was similar to the current study, and they measured postural control via motion capture and posture platform at four timepoints. Deficits in postural control were recorded to appear shortly after heading [20]. Postural control changes were also reported by Caccese et al. [16] with an increase in sway velocity in the youth, high school, and collegiate populations (aged 12–14, 15–18, and 18–22 years, respectively). Postural control was measured using the same MobileMat as our study, and they measured center of pressure over a two-minute time span. The heading protocol was nearly the same as the current study with one or two differences [16]. In recent years, a normative data set for pediatrics has been released for the SWAY Balance application [22]. The values we report for the tandem left stance and the single-leg right stance are within 1 to 2 standard deviations of the normative values for those aged 10–12 years. The 30% changes in the tandem left and single-leg right stances may be due to measurement errors as the standard deviations are quite large. Another possible cause of balance changes could be due to physical changes which affect the mechanisms of postural control that are still maturing during adolescence [23]. Even though the feet together stance had a significant difference, it is not clinically significant as a 2% difference has no clinical meaning.

BESS scores appeared to not be affected by an acute bout of heading in our sample of female youth soccer players. Similar results were reported in female collegiate soccer players (aged 18–21 years) after an acute bout of heading, in which the heading protocol was nearly the same as ours [15]. They reported no changes in BESS scores from pre- to post-heading in groups of various concussion histories and a control group. The number of headers during a season did not affect balance in female high school and collegiate soccer players aged 14–24 years [24]. The change in BESS scores from pre- to post-season was not correlated to the number of headers performed in games during the season even though the collegiate players headed the ball significantly more per game than the high school players. The BESS scores that we report are similar to normative values which have been reported for this age group previously [25,26]. BESS scores were reported to improve significantly with age from 9 years old to 18 years old [26]. The scores were approximately 26 and 25 respectively in the reported normative values compared to the score of 30 in the current study [25,26]. It is important to note that the normative values were computed by a human rater whereas our values were computer-derived. These two methods were previously compared and the computer-derived results were deemed to be comparative to the human rater results [27].

Similar to the results here, no change in self-reported symptoms and neurocognitive functioning have also been reported in previous studies [15,28,29]. Kaminski et al. [15] observed no change in neurocognitive functioning following an acute bout of heading in female collegiate soccer players aged 18–21 years. They utilized the same neurocognitive computerized test as we did, the ImPACT test. It was also reported that the soccer players with no concussion history had little to no change in self-reported symptoms pre- to post-heading [15]. Expanding the acute bout of heading to a full season of accumulating headers, Kontos et al. [28] reported that there was no relationship between the number of headers performed and the outcomes of neurocognitive functioning and self-reported symptoms in a group of youth and interscholastic male and female soccer players aged 13–18 years. This group also used the ImPACT test to assess neurocognitive function. In this study, the neurocognitive testing was only completed at the end of the season and was compared to existing ImPACT normative data. Female interscholastic soccer players aged 13–19 years old showed no difference in self-reported symptoms nor neurocognitive functioning from the start to the end of their interscholastic career (about 4 years) [29]. Self-reported symptom scores had little to no change over the course of 4 years. This study utilized a different neurocognitive function test called the Automated Neuropsychological Assessment Metrics (ANAM), which is now more commonly used in the military. Even though most of the previous studies were conducted in an older population than the current study, the results still support what we observed in our cohort of youth soccer players: no changes in self-reported symptoms or neurocognitive functioning related to an acute bout of heading.

Although we expected to see beneficial changes in head impact biomechanics following the intervention, we observed no change in PLA, PRA, or PRV. The values reported here are similar to those reported in female youth players during game situations [30,31]. Harriss et al. [30] used the GForce Tracker, similar to the Triax SIM-G, it is positioned at the back of the head via headband, to record head impact kinematics during games over the course of a season. The female soccer players (aged 12–14 years) in their study experienced a mean PLA of 18.8*g* compared to our value of about 23*g*. Hanlon et al. [31] measured head impact kinematics in the female U14 age group using the Head Impact Telemetry System during scrimmages. Similar to the current study, the average PLA was in the low 20*g* range. A few other studies have reported higher values at 30*g* in youth, high school, and collegiate male and female players in game scenarios and an acute heading protocol [12,32,33]. All of these studies used the same head impact sensor as we did to measure head impact kinematics, the Triax SIM-G. Caccese et al. [12,32] had participants complete an acute bout of heading which resulted in an average PLA of 35*g* in youth through collegiate soccer players. In just the youth players (aged 12–14 years), the average PLA was in the high 30*g* range [32]. Lamond et al. [33] observed collegiate female soccer players aged 18–21 years over the course of a season. The combined average PLA of games and practices was 28*g*. Even with no change in head impact biomechanics, the coaches and researchers observed an improvement in heading technique and form. The Get aHEAD Safely in Soccer™ program emphasizes the teaching of correct form and technique of purposeful heading through simple header drills. The current study utilized lightweight, size 5 “Header Trainer” soccer balls during the acute heading protocol and during the season for practice header drills. If a regulation size 5 soccer ball would have been used, it would have simulated game play headers more closely. However, we believe the lightweight soccer balls are safer and allow youth soccer players to safely practice their heading skills. Lightweight soccer balls should be implemented into header drills in the youth population to encourage safe heading technique and to build confidence when heading the ball.

We were limited to one female youth soccer team from the mid-Atlantic area of the United States resulting in a small sample size. A larger sample size would increase the accuracy of the data and decrease the variability. We were also limited to one fall soccer season which may have impacted the effectiveness of the Get aHEAD Safely in Soccer™ program. Another limitation is that we did not have a control group for comparison. However, we did baseline measures for the participants to serve as their own controls.

Future research should include the use of the Get aHEAD Safely in Soccer™ program over a longer period of time. One time frame of interest would be from when players are 11 years old to when they turn 13 years old, the age range in which US Soccer recommends limiting practice headers. Future research should also include a larger sample size, different age groups, and male players. In a different direction, researchers could explore the biomechanics of RHI using a finite element model of a human head as is done in impacts and blasts resulting in traumatic brain injuries [34].

## 5. Conclusions

Subtle balance changes from baseline to post-season were observed after an acute bout of heading. No changes in BESS scores, self-reported symptoms, neurocognitive functioning, or head impact kinematics were observed. Although there were no changes in head impact kinematics, coaches and researchers noted an improvement in heading technique and form. Utilizing lightweight soccer balls during training for header drills would be a safe and beneficial tool in the youth population.

## Figures and Tables

**Table 1:** Get aHEAD Safely in Soccer™ neck and core strengthening exercise progression.

		Week 1/2	Week 3/4	Week 5/6	Week 7/8	Week 9/10	Week 11/12
	Exercises	Sets/Reps	Sets/Reps	Sets/Reps	Sets/Reps	Sets/Reps	Sets/Reps
Day 1	4-way neck exercises (manual resistance)	1/10	1/10	1/10	1/10	1/10	1/10
	Shoulder Shrugs	1/15	1/20	1/20	1/20	1/20	1/20
	Partner Sit-Ups	1/15	1/20	1/25	1/25	1/35	1/50
	Hip Bridges	--	--	1/5	1/5	1/5	1/10
	Bird Dogs	--	--	--	1/5 each side	1/5 each side	1/5 each side
	Side Plank	--	--	--	--	1/5 each side	1/5 each side
	Lunges	--	--	--	--	--	1/5 each side
Day 2	4-way neck exercises (manual resistance)	1/10	1/10	1/10	1/10	1/10	1/10
	Shoulder Shrugs	1/15	1/20	1/20	1/20	1/20	1/20
	Partner Sit-Ups	1/15	1/20	1/25	1/25	1/35	1/50
	Hip Bridges	--	--	1/5	1/5	1/5	1/10
	Bird Dogs	--	--	--	1/5 each side	1/5 each side	1/5 each side
	Side Plank	--	--	--	--	1/5 each side	1/5 each side
	Lunges	--	--	--	--	--	1/5 each side

**Table 2:** Participant Demographics, Player Position, and Concussion History.

Participants (*n* = 12)	
Age (years)	10.5 ± 0.5
Height (cm)	148.6 ± 6.0
Weight (kg)	39.4 ± 7.2
Position	
Forward	2
Midfield	4
Defense	5
Goalkeeper	1
Concussion History	
0	12
≥1	0

Mean ± Standard Deviation.

**Table 3:** Baseline, pre-season, and post-season balance, neurocognitive, and head impact kinematic measures.

	Baseline	Pre-Season	Post-Season
SWAY (*n* = 12)			
Feet Together (%)	98.9 ± 1.9	99.0 ± 1.2	96.8 ± 3.0
Tandem Right (%)	88.0 ± 15.1	86.2 ± 13.6	73.7 ± 27.7
Tandem Left (%)	94.3 ± 5.6	88.3 ± 11.6	63.9 ± 31.9
Single-leg Right (%)	72.1 ± 21.8	54.6 ± 29.5	41.4 ± 36.1
Single-leg Left (%)	59.6 ± 27.1	53.5 ± 30.8	37.5 ± 38.2
Reaction Time (ms)	305.1 ± 73.8	299.2 ± 59.7	261.7 ± 46.8
Total (%)	73.3 ± 9.5	70.3 ± 9.1	66.3 ± 10.2
BESS (*n* = 12)	31.1 ± 8.2	30.0 ± 6.1	31.3 ± 5.9
ImPACT (*n* = 12)			
Verbal Memory	79.8 ± 15.5	87.9 ± 6.9	86.2 ± 12.1
Visual Memory	70.2 ± 18.8	71.1 ± 13.9	74.2 ± 16.3
Visual Motor Speed	28.3 ± 5.4	29.1 ± 6.2	30.1 ± 7.7
Reaction Time	0.79 ± 0.08	0.82 ± 0.13	0.78 ± 0.10
Symptom Score	1.8 ± 2.6	1.1 ± 1.7	1.7 ± 2.5
Head Impact Kinematics (*n* = 8)			
PLA (*g*)	--	22.6 ± 4.9	24.7 ± 11.4
PRA (krad/s^2^)	--	2.7 ± 1.3	2.4 ± 1.3
PRV (rad/s)	--	12.1 ± 3.6	10.7 ± 5.5

Mean ± Standard Deviation; Peak Linear Acceleration (PLA), Peak Rotational Acceleration (PRA), Peak Rotational Velocity (PRV).

## Data Availability

The data presented in this study are available on request from the corresponding author. The data are not publicly available due to local UDIRB restrictions.
